# Supplementary material for: Conservation and diversity in expression of candidate genes regulating socially-induced female-male sex change in wrasses
Source: PeerJ. 2019 Jun 11;7:e7032. doi: 10.7717/peerj.7032 (PMC6568253; doi:10.7717/peerj.7032)
Supplement: Table S1 — Abbreviations: NBF, non-breeding female; BF, breeding female; ET, early transitional; MT, mid transitional; LT, late transitional; TP, terminal phase, IP, initial phase. [file peerj-07-7032-s001.docx]

| **Stage** | **Description** |
| --- | --- |
| BF | Presence of vitellogenic or mature oocytes. No evidence of male structure. |
| NBF | Predominantly pre-vitellogenic oocytes, few atretic oocytes present. Lacks healthy vitellogenic oocytes. |
| ET | Atretic oocytes and nests of gonial cells common. Cell debris, which may also be present as yellow-brown bodies, and stromal cells often evident. No evidence of male structure. |
| MT | Oocyte numbers diminished and mostly atretic. Proliferation of spermatogonia evident. |
| LT | Number of spermatogenic cysts predominates over oocytes. More structured arrangement of cysts into lobules. Some atretic oocytes (mostly previtellogenic) may be present. |
| TP male | Presence of spermatozoa (seasonal) and/or lobules. Formation of peripheral sperm collection ducts. Evidence of being formerly female, e.g. presence of a lumen. |
| IP male | Presence of spermatozoa and/or lobules. Lacks evidence of having been a functional female, may have central collection ducts instead of a lumen. |
