# Supplementary material for: Conservation and diversity in expression of candidate genes regulating socially-induced female-male sex change in wrasses
Source: PeerJ. 2019 Jun 11;7:e7032. doi: 10.7717/peerj.7032 (PMC6568253; doi:10.7717/peerj.7032)
Supplement: Table S2 [file peerj-07-7032-s002.docx]

|  | **Tissue homogenisation** | **RNA extraction** | **DNase treatment** | **Reverse Transcription** |
| --- | --- | --- | --- | --- |
| *Bluehead Wrasse* |  |  |  |  |
| Gonad and  Brain (fore/midbrain) | TissueLyser II (Qiagen). | TRIzol reagent (Invitrogen).  Bromo-chloropropane (gonad) and chloroform (brain) for phase separation. Clean-up through NucleoSpin RNA XS columns (Macherey-Nagel). | TURBO DNA-free kit (Ambion) | 500 ng - PrimeScript RT reagent kit (Perfect Real Time) (Takara) with Oligo dT and random hexamer primers. |
| *Spotty Wrasse: Socially induced* |  |  |  |  |
| Gonad | Pestle mixer (Argos Technologies). | TRIzol (Invitrogen) | TURBO DNA-free kit (Ambion) | 900 ng - High Capacity cDNA RT kit (Applied Biosystems) with random hexamer primers. |
| Brain (fore/midbrain) |  |  |  | 1,000 ng - High Capacity cDNA RT kit (Applied Biosystems) with random hexamer primers. |
| *Spotty Wrasse: Opportunistic* |  |  |  |  |
| Gonad and  Brain (fore/midbrain) | Needle and syringe. | PureLink RNA Mini Kit (ThermoFisher Scientic). | TURBO DNA-free kit (Ambion). | 500 ng - High Capacity cDNA RT kit (Applied Biosystems) with random hexamer primers. |
| *Kyusen Wrasse* |  |  |  |  |
| Gonad and  Brain (whole) | Pestle mixer (Argos Technologies). | DNA/RNA dual extraction ZR-Duet™ DNA/RNA MiniPrep (Zymo). | On-column RNAse-free DNase I Kit (Norgen). | 500 ng - High Capacity cDNA RT kit (Applied Biosystems) with random hexamer primers. |
