# Supplementary material for: Conservation and diversity in expression of candidate genes regulating socially-induced female-male sex change in wrasses
Source: PeerJ. 2019 Jun 11;7:e7032. doi: 10.7717/peerj.7032 (PMC6568253; doi:10.7717/peerj.7032)
Supplement: Table S3 — Bluehead primers were used to determine partial gene sequences for kyusen wrasse. Abbreviations: 18S, 18S ribosomal RNA, amh, anti-müllerian hormone, cyp19a1a, gonadal aromatase, cyp19a1b, brain aromatase, ef1a, elongation factor 1 α, FW, forward, g6pd, glucose-6-phosphate dehydrogenase, it, isotocin, RV, reverse, Tm, melting temperature. [file peerj-07-7032-s003.docx]

| **Gene** | **Species** | **Primer** | **Tm (^O^C)** | **Amplicon size (bp)** |
| --- | --- | --- | --- | --- |
| *cyp19a1a* | bluehead, spotty, kyusen | FW: GGCAGACTGTGCTCATCAAA | 60 | 690 |
|  |  | RV: GCAGAGTCACCAGGATGGAT | 60.1 |  |
| *amh* | bluehead, kyusen | FW: ACGCCTCATCAACAAAAACC | 60 | 685 |
|  |  | RV: AGCTCACACAGGAAGGAGGA | 60 |  |
|  | spotty | FW: CTTTGTGGACGATGTGGTTG | 60 | 687 |
|  |  | RV: TCAAGCTGAAGGGAAGAGGA | 60.1 |  |
| *cyp19a1b* | bluehead, spotty, kyusen | FW: GTGGGAATCTGTGTGAGCWC | 55.8 | 710 |
|  |  | RV: TTCAGAATGATGTTGGTTCCTTTTG | 53.9 |  |
| *it* | bluehead, kyusen | FW: GTGTCCGTGTGCCTTCTTTT | 60.1 | 641 |
|  |  | RV: TGCAGATATGTGGATGACCA | 58.4 |  |
|  | spotty | FW: GTGTCCGTGTGCCTTCTTTT | 60.2 | 634 |
|  |  | RV: TCTGGATGACCATAGTCAGTATTCA | 59.9 |  |
| *ef1a* | bluehead, spotty, kyusen | FW: TGCGGWGGAATCGACAAGAG | 57.2 | 699 |
|  |  | RV: CAACACCAGCAGCAACAATC | 57.5 |  |
| *18S* | bluehead, spotty, kyusen | FW: GAAATTCTTGGACCGGCGC | 57.2 | 700 |
|  |  | RV: TTATGACCCGCGCTTACTGG | 57.3 |  |
| *g6pd* | bluehead, spotty,  kyusen | FW: CCTCATGGTBCTCAGRTTTGG | 56 | 735 |
|  |  | RV: CTGCGHACRAAGTGCATCTG | 56.4 |  |
