# Supplementary material for: Conservation and diversity in expression of candidate genes regulating socially-induced female-male sex change in wrasses
Source: PeerJ. 2019 Jun 11;7:e7032. doi: 10.7717/peerj.7032 (PMC6568253; doi:10.7717/peerj.7032)
Supplement: Table S4 — For spotty wrasse the average efficiency ± standard deviation of all the qPCR plates for one gene is shown. Abbreviations: 18S, 18S ribosomal RNA; amh, anti-müllerian hormone; bp, base pairs; cyp19a1a, gonadal aromatase, cyp19a1b, brain aromatase; ef1a, elongation factor 1α; FW, forward; g6pd, glucose-6-phosphate dehydrogenase; it, isotocin; n/a, not applicable, RV, reverse. [file peerj-07-7032-s004.docx]

| **Gene** | **Primer** | **Annealing temp. (^O^C)** | **Amplicon size (bp)** | **Efficiency (%) in gonad samples** | **Efficiency (%) in brain samples** |
| --- | --- | --- | --- | --- | --- |
| Bluehead wrasse | | | | | |
| *cyp19a1a* | FW: ACCAGAAGCACAAGATGGCA | 56.5 | 146 | 99.3 | n/a |
|  | RV: GCTCACCATGGTTCTGAGCA |  |  |  |  |
| *amh* | FW: AGCTCTCATCAGAGGGAGGTG | 56.5 | 161 | 100.6 | n/a |
|  | RV: GGGTTGTAGTTCATGGCTTGTG |  |  |  |  |
| *cyp19a1b* | FW: GCTCATCTTTGCTCAGAACCA | 56.5 | 168 | n/a | 99.5 |
|  | RV: TTCATCTCCTCCACTATCCTCAA |  |  |  |  |
| *it* | FW: ACCACAGCGCAAGTGCAT | 56.5 | 148 | n/a | 92.6 |
|  | RV: ACAGGGGGTGAGCAGATAGTT |  |  |  |  |
| *ef1a* | FW: GTGGAAGTTTGAGACCAGCAA | 62 | 131 | 100 | 95.9 |
|  | RV: CAACACCAGCAGCAACAATC |  |  |  |  |
| *18S* | FW: GGTTAATTCCGATAACGAACGA | 65 | 143 | 98.4 | 95.3 |
|  | RV: ACATCTAAGGGCATCACAGACC |  |  |  |  |
| *g6pd* | FW: AATAATTCGCGACGTCATGC | 61.5 | 104 | 99.5 | 99.1 |
|  | RV: CCTTTTCATCCCTGACATCATC |  |  |  |  |
| Spotty wrasse | | | | | |
| *cyp19a1a* | FW: CCAGGCCCGTCTTTCTATCT | 62.5 | 149 | 98.8±1.2 | n/a |
|  | RV: AGTATGAGCGTCTCCTCTCCATT |  |  |  |  |
| *amh* | FW: TGTTGGTGACAATGGTGAACTC | 62.5 | 158 | 94.7±1.0 | n/a |
|  | RV: GCTCTCCAGATAGATGCCAAAC |  |  |  |  |
| *cyp19a1b* | FW: AGATCTGCAGGACGTGATGG | 62.5 | 102 | n/a | 98.6±1.0 |
|  | RV: ATGAGCTCTGTTGCAAAGTCAAG |  |  |  |  |
| *it* | FW: CGCAAGTGCATGTCGTGT | 62.5 | 112 | n/a | 96.8±1.7 |
|  | RV: CGCAGTGAGCTGTTTCTGG |  |  |  |  |
| *ef1a* | FW: GCAAGGTACTACGTGACCATCA | 62.5 | 128 | 98.6±0.9 | 95.4±2.3 |
|  | RV: GCCTCAAACTCACCAACACC |  |  |  |  |
| *g6pd* | FW: CGACGTCATGCAGAACCA | 62.5 (gonad) | 106 | 98.3±1.2 | 94.8±1.6 |
|  | RV: CAGCACCTTCACCTTTTCGT | 60 (brain) |  |  |  |
| *18S* | FW: GGTTAATTCCGATAACGAACGA | 62.5 | 143 | 94.9±1.8 | 95.9±2.4 |
|  | RV: ACATCTAAGGGCATCACAGACC |  |  |  |  |
| Kyusen wrasse | | | | | |
| *cyp19a1a* | FW: CCTGGATGTTCCTGTGAATGA | 60 | 191 | 99.9 | n/a |
|  | RV: CCACCTCTCTCCTCTTCTGCT |  |  |  |  |
| *amh* | FW: CAGCAGCCTCGGTCTTAACA | 50 | 175 | 98.6 | n/a |
|  | RV: AGCAGCACAGGGTTTGACTT |  |  |  |  |
| *cyp19a1b* | FW: TGGCTCTACAACAAGCACAAGA | 60 | 200 | n/a | 96.5 |
|  | RV: ATCACCATCTCCAGCACACAC |  |  |  |  |
| *it* | FW: GATCTGAGGGAGGACGCTGT | 60 | 111 | n/a | 99.3 |
|  | RV: ATTGGCTGGTTGGGTCGT |  |  |  |  |
| *ef1a* | FW: GGCCCGTTTTGAGGAAATC | 57.5 | 161 | 93.8 | 98.7 |
|  | RV: GCTCAATCTTCCATCCCTTG |  |  |  |  |
| *g6pd* | FW: ACGAGGAGGATACTTTGACGATT | 57.5 | 148 | 100.3 | 99 |
|  | RV: ACACTTCAGCACCTTCACCTTT |  |  |  |  |
| *18S* | FW: TTAATTCCGATAACGAGCGAGA | 57.5 | 141 | 92 | 95.3 |
|  | RV: ACATCTAAGGGCATCACAGACC |  |  |  |  |
