# Supplementary material for: Conservation and diversity in expression of candidate genes regulating socially-induced female-male sex change in wrasses
Source: PeerJ. 2019 Jun 11;7:e7032. doi: 10.7717/peerj.7032 (PMC6568253; doi:10.7717/peerj.7032)
Supplement: Table S5 [file peerj-07-7032-s005.docx]

| *Item* *to* *Check* | *Importance* | *Checklist* |
| --- | --- | --- |
| Experimental Design | | |
| Definition of experimental and control groups | E | Bluehead wrasse: Experimental groups: Stages 1 – 6, TP male, IP male. Control group: female.  Spotty, socially induced: Experimental groups: C BF, M BF, ET, MT, LT, TP male, IP male. Control group: C BF D0.  Spotty, opportunistic sampling: Experimental groups: ET, MT, LT, TP male. Control group: NBF  Kyusen wrasse: Experimental groups: ET, TP male, IP male. Control group: NBF. |
| Number within each group | E | Gonads: Bluehead wrasse n = 3 all groups, spotty wrasse socially induced to change sex in captivity C BF D0 n = 5, C BF n = 16, M BF n = 4, ET n = 20, MT n = 1, LT n = 1, TP n = 11, IP n = 5, spotty wrasse opportunistically caught NBF n = 6, ET n = 3, MT n = 2, LT n = 2, TP n = 1, kyusen wrasse NBF n = 7, ET n = 3, TP n = 11, IP n = 3  Brains: Bluehead wrasse n = 3 all groups, spotty wrasse socially induced to change sex in captivity C BF D0 n = 5, C BF n = 16, M BF n = 4, ET n = 20, MT n = 1, LT n = 1, TP n = 11, IP n = 5, spotty wrasse opportunistically caught NBF n = 6, ET n = 2, MT n = 2, LT n = 3, TP n = 1, kyusen wrasse NBF n = 6, ET n = 5, TP n = 7, IP n = 4 |
| Assay carried out by core or investigator’s laboratory? | D | Investigator’s laboratory |
| Acknowledgement of authors contributions | D | Yes, see Contributions |
| Sample | | |
| Description | E | Bluehead wrasse: one gonadal lobe, spotty socially induced: mid-section of one gonadal lobe, spotty opportunistically caught: one gonadal lobe, kyusen wrasse gonad section.  One forebrain/midbrain from each fish, except kyusen wrasse whole brain. |
| Volume/mass of sample processed | D | - |
| Microdissection or macrodissection | E | Macrodissection |
| Processing procedure | E | See methods: Sample Collection. |
| If frozen, how and how quickly? | E | Bluehead wrasse: preserved in RNAlater (Life Technologies, Inc.) on ice then transferred to -20°C.  Spotty socially induced: preserved in RNAlater (Invitrogen ^TM^, Thermo Fisher Scientific) then transferred to -20°C.  Spotty opportunistic: preserved in RNAlater (Life Technologies, Inc.) on ice, then transferred to -80°C.  Kyusen: preserved in RNA later (Life Technologies, Inc.) on ice, or flash frozen in liquid nitrogen, then transferred to -80^O^C. |
| If fixed, with what and how quickly? | E | Not fixed |
| Sample storage conditions and duration | E | -80°C until RNA extraction |
| Nucleic Acid Extraction | | |
| Procedure and/or instrumentation | E | See Methods, Table S2. |
| Name of kit and details of any modifications | E | See Methods, Table S2. |
| Source of additional reagents used | D | TRIzol Reagent (Invitrogen), BCP (Sigma-Aldrich) |
| Details of DNase or RNase treatment | E | See Methods, Table S2. |
| Contamination assessment (DNA or RNA) | E | No reverse transcription control done |
| Nucleic acid quantification | E | Qubit 2.0 Fluorometer (Qubit RNA HS Assay Kit, Life Technologies) |

| Yield | D | > 1.2 µg |
| --- | --- | --- |
| Instrument and method | E | Qubit 2.0 Fluorometer (Qubit RNA HS Assay Kit, Life Technologies) |
| Purity(A_260_/A_280_) | D | A_260_/A_280_ = 1.8 – 2.1 |
| RNA integrity: method/instrument | E | Bluehead wrasse RNA assessed on an Agilent 2100 Bioanalyzer. Others not. |
| RIN/RQI or Cq of 3’ and 5’ transcripts | E | Bluehead wrasse RIN = 7.3 – 9.6 |
| Electrophoresis traces | D | Yes |
| Inhibition testing (Cq dilutions, spike, or other) | E | Not performed, PrimeScript RT reagent kit (Perfect Real Time) (Takara) contains Tli RNase H which minimises PCR inhibition |
| **Reverse Transcription** | | |
| Complete Reaction Conditions | E | See Methods, Table S2 and Methods, RNA extraction. |
| Amount of RNA and reaction volume | E | See Methods, Table S2 |
| Priming oligonucleotide (if using GSP) and concentration | E | N/A |
| Reverse transcriptase and concentration | E | Bluehead wrasse: PrimeScript RT Enzyme Mix I , concentration not stated  All others: MultiScribe™ Reverse  Transcriptase 2.5U/µL |
| Temperature and time | E | Bluehead wrasse: 37°C (15 minutes), 85°C (5 seconds), 4°C until removal .  All others: 25 ^O^C (10 mins), 37 ^O^C (120 min), 85 ^O^C (5 min), 4 ^O^C until removal. |
| Manufacturer of reagents and catalogue numbers | D | - |
| Cqs with and without reverse transcription | D | - |
| Storage conditions of cDNA | D | Stored at -70°C |
| **qPCR Target Information** | | |
| Gene symbol | E | *cyp19a1a, amh, cyp19a1b, it*  reference genes = *ef1a, 18S, g6pd* |
| Sequence accession number | E | Bluehead wrasse:  *cyp19a1a* MK252274*, amh* MK252275*, cyp19a1b* MK252276*, it* MF279538*.1, g6pd* MK252277*, ef1a* MF279537.1, *18S* MK246126*,* spotty wrasse *cyp19a1a* MK252278*, amh* MK252279*, cyp19a1b* MK252280*, it* MK252281*, g6pd* MK252282*, ef1a* MK252283*, 18S* MK246127*,* kyusen wrasse *cyp19a1a* MK252284*, amh* MK252285*, cyp19a1b* MK252286*, it* MK252287*, g6pd* MK252288*, ef1a* MK252289*, 18S* MK246128*.* |
| Location of amplicon | D | Bluehead wrasse:  *cyp19a1a:* 36 – 182 bp, *amh*: 247 – 407 bp, *cyp19a1b*: 344 – 511 bp  , *it*: 81 – 228 bp, *ef1a*: 126 - 256 bp, *18S*: 391 – 533 bp, *g6pd*: 112 – 215 bp.  Spotty wrasse:  *cyp19a1a:* 160-308 bp, *amh*: 31 – 188 bp, *cyp19a1b*: 256 – 357 bp  , *it*: 88-199 bp, *ef1a*: 144 – 271 bp, *18S*: 392 – 534 bp, *g6pd*: 114-219 bp.  Kyusen wrasse:  *cyp19a1a:* 475 – 597 bp, *amh*: 1 – 178 bp, *cyp19a1b*: 1 – 201 bp  , *it*: 252 – 362 bp, *ef1a*: 390 – 550 bp, *18S*: 99 - 239 bp, *g6pd*: 85 - 232 bp. |
| Amplicon length | E | See Methods, Table S4. |
| In silico specificity screen (BLAST, and so on) | E | - |
| Pseudogenes, retropseudogenes, or other homologs? | D | - |
| Sequence alignment | D | The qPCR products were visualised using electrophoresis through a 1% agarose gel using SYBR Safe DNA Gel Stain (Invitrogen). The amplicons were extracted from the gel using a NucleoSpin gel and PCR clean-up kit (Macherey-Nagel) and sanger sequenced in sense and antisense directions using the respective PCR primers by the Genetic Analysis Services, Department of Anatomy and Structural Biology, University of Otago. The qPCR amplicon sequences were aligned with the respective transcript in CLC Sequence Viewer 7 (Qiagen). This confirmed the qPCR products were specific. |
| Secondary structure analysis of amplicon | D | - |
| Location of each primer by exon or intron (if applicable) | E | - |
| What splice variants are targeted? | E | - |
| **qPCR Oligonucleotides** | | |
| Primer sequences | E | See Methods, Table S4. |
| RT Primer DB identification number | D | - |
| Probe sequences | D | - |
| Location and identity of any modifications | E | N/A |
| Manufacturer of oligonucleotides | D | Integrated DNA Technologies (NSW, Australia) |
| Purification method | D | Standard desalting |
| Complete reaction conditions | E | See below |
| Reaction volume and amount of cDNA/DNA | E | Reaction volume: 10 µL, Amount of cDNA: 20 ng (except *18S* 0.2 ng) |
| Primer, (probe), Mg^2+^, and dNTP concentrations | E | Primer concentration: 1 µM  Mg^2+^, and dNTP mixture contained in SYBR^®^ Premix Ex Taq^TM^II (Tli RNaseH Plus) (Takara), concentrations unknown |
| Polymerase identity and concentration | E | SYBR^®^ Premix Ex Taq^TM^(Tli RNaseH Plus) (Takara) contains Ex Taq HS DNA polymerase (Takara), concentration unknown |
| Buffer/kit identity and manufacturer | E | SYBR^®^ Premix Ex Taq^TM^II (Tli RNaseH Plus) (Takara) |
| Exact chemical composition of buffer | D | Unknown |
| Additives (SYBR Green I, DMSO, and so forth) | E | SYBR^®^ Premix Ex Taq^TM^II (Tli RNaseH Plus) (Takara) contains SYBR Green I |
| Manufacturer of plates/tubes and catalogue number | D | MicroAmp® optical 96-well reaction plate (Life Technologies)  Catalogue Number: N8010560 |
| Complete thermocycling parameters | E | 95°C for 2 minutes followed by 40 cycles of 95°C for 5 seconds, annealing temperature (see Methods, Table S4) for 10 seconds, and 72°C for 5 seconds. After 40 cycles, the melting curve analysis was run with cycling conditions of 95°C for 1 minute, 55°C for 30 seconds and 95°C for 30 seconds |
| Reaction setup (manual/robotic) | D | Manual |
| Manufacturer of qPCR instrument | E | QuantStudio 5 Real-Time PCR system (ThermoFisher) |
| **qPCR Validation** | | |
| Evidence of optimisation (from gradients) | D | Trialed primers at annealing temperatures of 50, 52.5, 55, 57.5, 60, 62.5, 65, 67.5 and 70°C |
| Specificity (gel, sequence, melt, or digest) | E | Melting curve analysis had a single peak.  Single amplicon confirmed in a 1% agarose gel electrophoresis stained with SYBR Safe DNA Gel Stain (Invitrogen).  Single band from the gel was extracted using a NucleoSpin gel and PCR clean-up kit (Macherey-Nagel) and sequenced in the sense and antisense directions by the Genetic Analysis Services.  No template controls were run for each gene to detect contamination and primer dimer formation. |
| For SYBR Green I, Cq of the NTC | E | Cq for all NTCs was >33 |
| Calibration curves with slope and y intercept | E | Bluehead wrasse:  *cyp19a1a:* y = -3.34 + 15.30  *amh:* y = -3.31 + 13.05  *cyp19a1b:* y *=* -3.34 + 12.55  *it:* y = -3.51 + 13.75  *ef1a* gonad: y = -3.32 + 13.08  *ef1a* brain*:* y = -3.42 + 13.05  *18S* gonad: y = -3.36 + 15.09  *18S* brain: y = -3.44 + 14.47  *g6pd* gonad: y = -3.34 + 12.23  *g6pd* brain: y = -3.34 + 12.90  Spotty wrasse socially induced (3 plates):  *cyp19a1a:* y = -3.39 + 13.37, -3.36 + 12.54, -3.31 + 13.24  *amh:* y = -3.44 + 14.11, -3.49 + 14.24, -3.47 + 14.17  *cyp19a1b:* y *=* -3.33 + 14.80, -3.39 + 15.03, -3.33 + 14.99  *it:* y = -3.35 + 16.08, -3.38 + 15.36, -3.42 + 15.45  *ef1a* gonad: y = -3.36 + 14.36, -3.38 + 14.85, -3.32 + 14.76  *ef1a* brain*:* y = -3.42 + 14.76, -3.54 + 14.51, -3.39 + 15.28  *18S* gonad: y = -3.45 + 14.67, -3.41 + 15.25, -3.41 + 14.62  *18S* brain: y = -3.33 + 16.23, -3.42 + 15.37, -3.50 + 15.64  *g6pd* gonad: y = -3.42 + 16.48, -3.34 + 15.25, -3.36 + 15.73  *g6pd* brain: y = -3.51 + 14.99, -3.41 + 16.10, -3.41 + 16.73  Spotty wrasse opportunistically caught:  *cyp19a1a:* y = -3.36 + 13.61  *amh:* y = -3.42 + 14.18  *cyp19a1b:* y *=* -3.38 + 14.84  *it:* y = -3.47 + 15.15  *ef1a* gonad: y = -3.36 + 14.64  *ef1a* brain*:* y = -3.40 + 14.37  *18S* gonad: y = -3.53 + 14.77  *18S* brain: y = -3.46 + 15.78  *g6pd* gonad: y = -3.34 + 16.06  *g6pd* brain: y = -3.48 + 14.95  Kyusen wrasse:  *cyp19a1a:* y = -3.34 + 7.31  *amh:* y = -3.36 + 12.05  *cyp19a1b:* y *= -*3.41 + 13.78  *it:* y = -3.34 + 14.50  *ef1a* gonad: y = -3.48 + 13.73  *ef1a* brain*:* y = -3.36 + 13.91  *18S* gonad: y = -3.53 + 13.99  *18S* brain: y = -3.44 + 13.87  *g6pd* gonad: y = -3.31 + 14.28  *g6pd* brain: y = -3.35 + 14.20 |
| PCR efficiency calculated from slope | E | See Methods, Table S4 |
| CIs for PCR efficiency or SE | D | Bluehead wrasse SE:  *cyp19a1a:* 0.04  *amh:* 0.05  *cyp19a1b:* 0.17  *it* = 0.03  *ef1a* gonad: 0.03  *ef1a* brain: 0.03  *18S* gonad: 0.04  *18S* brain: 0.09  *g6pd* gonad: 0.06  *g6pd* brain: 0.03  Spotty wrasse socially induced (3 plates) SE:  *cyp19a1a:* 0.03, 0.04, 0.03  *amh:* 0.04, 0.05, 0.03  *cyp19a1b:* 0.09, 0.08, 0.07  *it* = 0.14, 0.06, 0.05  *ef1a* gonad: 0.03, 0.03, 0.05  *ef1a* brain: 0.06, 0.06, 0.12  *18S* gonad: 0.04, 0.09, 0.03  *18S* brain: 0.18, 0.10, 0.07  *g6pd* gonad: 0.25, 0.17, 0.15  *g6pd* brain: 0.06, 0.18, 0.17  Spotty wrasse opportunistically caught SE:  *cyp19a1a:* 0.02  *amh:* 0.09  *cyp19a1b:* 0.08  *it:* 0.13  *ef1a* gonad: 0.03  *ef1a* brain*:* 0.05  *18S* gonad: 0.02  *18S* brain: 0.12  *g6pd* gonad: 0.18  *g6pd* brain: 0.11  Kyusen wrasse SE:  *cyp19a1a*: 0.04  *amh:* 0.05  *cyp19a1b:* 0.02  *it*: 0.07  *ef1a* gonad: 0.02  *ef1a* brain: 0.02  *18S* gonad: 0.02  *18S* brain: 0.17  *g6pd* gonad: 0.05  *g6pd* brain: 0.04 |
| r^2^of calibration curve | E | All genes in bluehead, spotty and kyusen wrasse: 1.0 |
| Linear dynamic range | E | - |
| C_q_variation at LOD | E | - |
| CIs throughout range | D | - |
| Evidence for LOD | E | - |
| If multiplex, efficiency and LOD of each assay | E | N/A |
| **Data Analysis** | | |
| qPCR analysis program (source, version) | E | QuantStudio Design and Analysis Software v1.2 (ThermoFisher) |
| Method of Cq determination | E | Threshold was determined automatically by the software, and the threshold was used to determine the Cq values of samples |
| Outlier identification and disposition | E | qPCR reactions that failed (no amplification) were excluded from analysis |
| Results for NTCs | E | Cq>33 for all NTCs |
| Justification of number and choice of reference genes | E | qPCR assays were carried out for 3 potential reference genes (*18S, g6pd, ef1a*). A non-parametric Kruskal-Wallis test, followed by post hoc comparisons using Dunns tests, with Benjamini Hochberg correction for multiple comparisons was done in R on the raw quantity data. Reference gene(s) showing no significant difference in distribution across sexes, and the flattest expression profile across sexes were chosen for each experiment. |
| Description of normalization method | E | The mean mRNA quantity of each sample was divided by that of the chosen references gene(s) (see Methods, Table S6), producing a normalized mRNA quantity for each sample. |
| Number and concordance of biological replicates | D | - |
| Number and stage (reverse transcription or qPCR) of technical replicates | E | qPCR reactions performed in duplicate (bluehead wrasse) and triplicate (all others) |
| Repeatability (intraassay variation) | E | - |
| Reproducibility (interassay variation, CV) | D | - |
| Power Analysis | D | - |
| Statistical methods for results significance | E | A non-parametric Kruskal-Wallis test, followed by post hoc comparisons using Dunns tests, with Benjamini Hochberg correction for multiple comparisons. |
| Software (source, version) | E | R |
| Cq or raw data submission with RDML | D | - |
