# Supplementary material for: Conservation and diversity in expression of candidate genes regulating socially-induced female-male sex change in wrasses
Source: PeerJ. 2019 Jun 11;7:e7032. doi: 10.7717/peerj.7032 (PMC6568253; doi:10.7717/peerj.7032)
Supplement: Table S6 — Three criteria were used to determine what potential reference genes would be chosen to normalise the results; sex did not significantly affect expression of the gene(s), the gene(s) showed a flat expression profile across sexes and normalisation did not change the overall trend of the results. In experiment 2 gonad samples, sex had a significant effect on expression of all potential reference genes. However, ef1a and 18S were chosen as reference genes because normalisation to these genes did not affect the trend of the results. In survey 1 gonad samples, g6pd was the only gene in which sex did not significantly affect expression. However, normalisation drastically changed the trend of the results. See Figs. 7E and 7F and Figs. S1E and S1F to compare un-normalised and normalised data, respectively. Abbreviations: 18S, 18S ribosomal RNA; ef1a, elongation factor 1 α; g6pd, glucose-6-phosphate dehydrogenase. [file peerj-07-7032-s006.docx]

| **Experiment/Survey** | **Tissue** | **Chosen Reference gene(s)** | **No significant effect of sex on expression** | **Flat expression profile across sexes** | **Normalisation with this gene(s) did not affect overall trend of results** |
| --- | --- | --- | --- | --- | --- |
| Experiment 1: Social induction of sex change in wild bluehead wrasse | Gonad | *18S* and *g6pd* | ✓ | ✓ | ✓ |
|  | Fore/midbrain | *ef1a* | ✓ | ✓ | ✓ |
| Experiment 2: Social induction of sex change in captive spotty wrasse | Gonad | *ef1a* and *18S* | 🗶 | 🗶 | ✓ |
|  | Fore/midbrain | *ef1a* and *g6pd* | ✓ | ✓ | ✓ |
| Survey 1: Opportunistic sampling of spotty wrasse | Gonad | *g6pd* | ✓ | ✓ | 🗶 |
|  | Fore/midbrain | *g6pd* | ✓ | ✓ | ✓ |
| Survey 2: Wild-caught kyusen wrasse | Gonad | *ef1a* and *g6pd* | ✓ | ✓ | ✓ |
|  | Whole brain | *ef1a* | ✓ | ✓ | ✓ |
