# Supplementary figures and images for: Conservation and diversity in expression of candidate genes regulating socially-induced female-male sex change in wrasses
Source: PeerJ. 2019 Jun 11;7:e7032. doi: 10.7717/peerj.7032 (PMC6568253; doi:10.7717/peerj.7032)

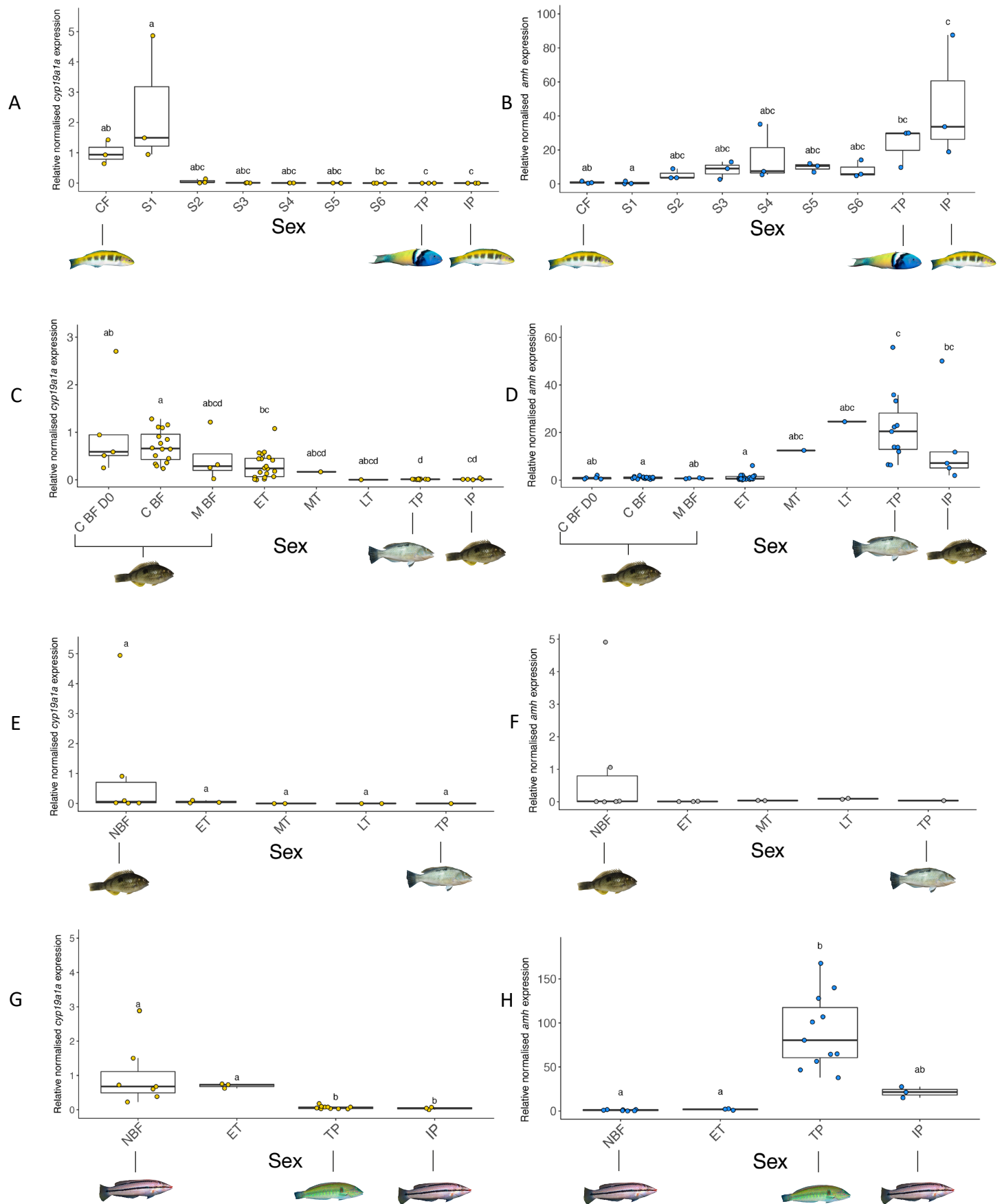

Supplement: Figure S1 — Expression levels are compared among females, transitioning fish, TP males and IP males. (A, B) Bluehead wrasse induced to change sex in the wild (Experiment 1). (C, D) Spotty wrasse induced to change sex in captivity (Experiment 2). (E, F) Wild-caught spotty wrasse (Survey 1). (G, H) Wild-caught kyusen wrasse (Survey 2). In survey 1 gonad samples (E, F), normalisation drastically changed the trend of the results. After normalisation both cyp19a1a and amh show the same expression profile; near zero expression of ET, MT, LT and TP fish. This indicates that the expression profile of the reference gene is overpowering that of cyp19a1a and amh. Points represent individual fish. Boxplots represent the median, lower and upper quartile values, and 1.5-fold the interquartile range. Yellow, blue and grey points indicate expression is significantly female-biased, male-biased, and non-significantly different, respectively. Letters denote a significant difference in distribution between groups and ‘a’ indicates overall significance without significant pairwise differences. Sample sizes: bluehead wrasse n = 3, all groups; spotty wrasse socially induced to change sex in captivity C BF D0 n = 5, C BF n = 16, M BF n = 4, ET n = 20, MT n = 1, LT n = 1, TP n = 11, IP n = 5; spotty wrasse opportunistically caught NBF n = 6, ET n = 3, MT n = 2, LT n = 2, TP n = 1; kyusen wrasse NBF n = 7, ET n = 3, TP n = 11, IP n = 3. Abbreviations: C BF D0, breeding female from control tank (TP male present) at experiment day 0; C BF, breeding female from control tank (TP male present) removed at progressive time points throughout the experiment; CF, control female; ET, early transitional; IP, initial phase male; LT, late transitional; M BF, breeding female from manipulated tanks (TP male removed) removed at progressive time points throughout experiment; MT, mid transitional; NBF, non-breeding female; S1-6, stages 1-6; TP, terminal phase male. See Fig. 1 legend for photo credits of female and male bl [file peerj-07-7032-s007.pdf]

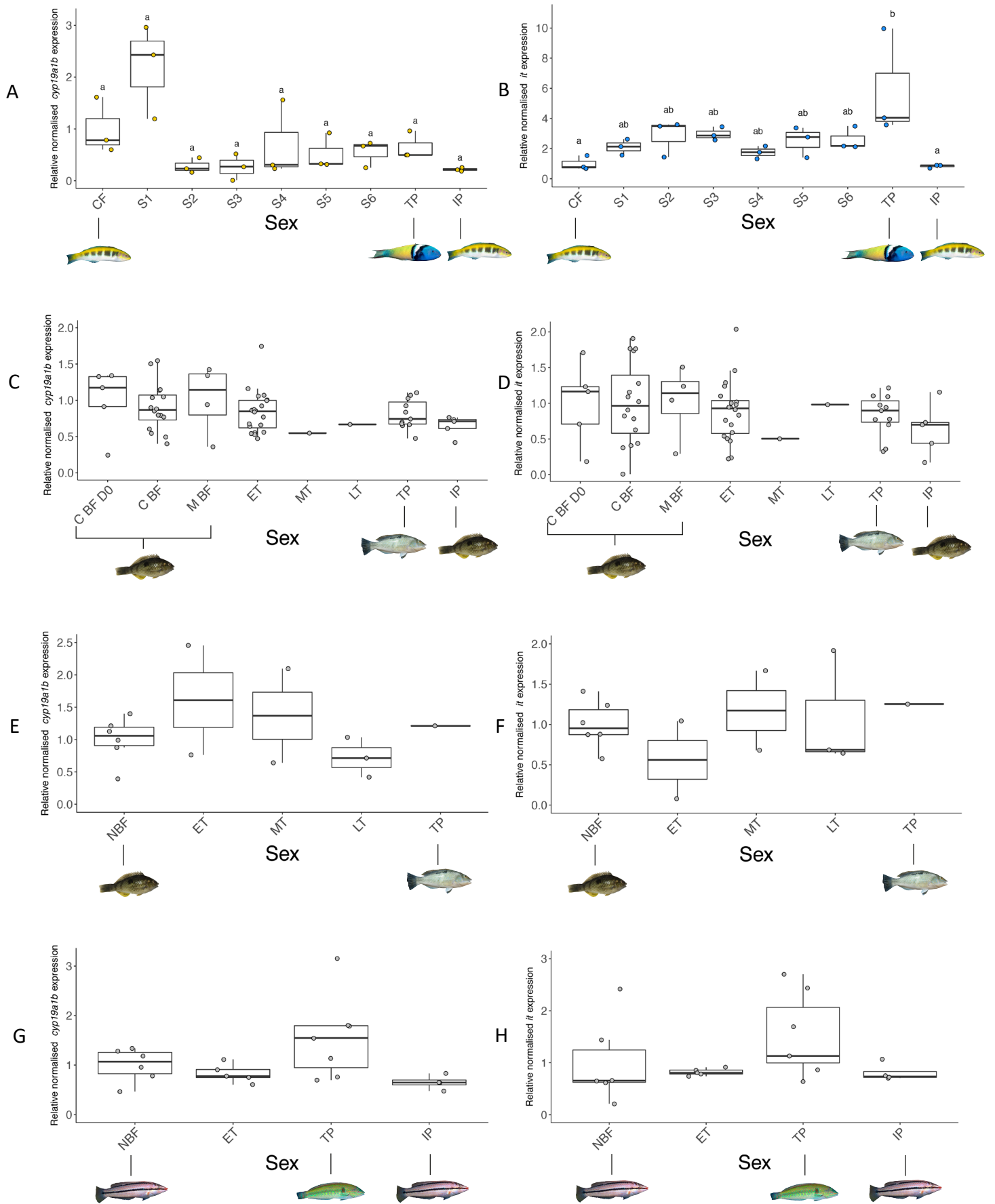

Supplement: Figure S2 — Expression levels are compared among females, transitioning fish, TP males and IP males. (A, B) Bluehead wrasse induced to change sex in the wild (Experiment 1). (C, D) Spotty wrasse induced to change sex in captivity (Experiment 2). (E, F) Wild-caught spotty wrasse (Survey 1). (G, H) Wild-caught kyusen wrasse (Survey 2). Points represent individual fish. Boxplots represent the median, lower and upper quartile values, and 1.5-fold the interquartile range. Yellow, blue and grey points indicate expression is significantly female-biased, male-biased, and non-significantly different, respectively. Letters denote a significant difference in distribution between groups and ‘a’ indicates overall significance without significant pairwise differences. Sample sizes: bluehead wrasse n = 3 all groups; spotty wrasse socially induced to change sex in captivity C BF D0 n = 5, C BF n = 16, M BF n = 4, ET n = 20, MT n = 1, LT n = 1, TP n = 11; IP n = 5, spotty wrasse opportunistically caught NBF n = 6, ET n = 2, MT n = 2, LT n = 3, TP n = 1; kyusen wrasse NBF n = 6, ET n = 5, TP n = 7, IP n = 4. Abbreviations: C BF D0, breeding female from control tank (TP male present) experimental day 0; C BF, breeding female from control tank (TP male present) removed at progressive time points throughout the experiment; CF, control female; ET, early transitional; IP, initial phase male; LT, late transitional; M BF, breeding female from manipulated tanks (TP male removed) removed at progressive time points throughout experiment; MT, mid transitional; NBF, non-breeding female; S1-6, stages 1-6; TP, terminal phase male. See Fig. 1 legend for photo credits of female and male bluehead, spotty and kyusen wrasses. [file peerj-07-7032-s008.pdf]
