# Supplementary material for: Conservation and diversity in expression of candidate genes regulating socially-induced female-male sex change in wrasses
Source: PeerJ. 2019 Jun 11;7:e7032. doi: 10.7717/peerj.7032 (PMC6568253; doi:10.7717/peerj.7032)
Supplement: Data S1 [file peerj-07-7032-s009.rtf]

###Example R code for statistical analysis and graphical illustration of qPCR data#NORMALISED#load dataK.gonads.raw <- read.csv("K.gonads.raw.csv", header=T)attach(K.gonads.raw)  #allows directly naming variablesnames(K.gonads.raw)  #gives overview of variable names in datasetK.gonads.raw$Group <- as.factor(K.gonads.raw$Group)#Kruskal Wallis testkruskal.test(Norm.Cyp19a1a.Eef1aG6pd~Group, data = K.gonads.raw)#Dunn test with BH correctionlibrary("dunn.test")library("FSA")dunnNorm.Cyp19a1a.Eef1aG6pd <- dunnTest(K.gonads.raw$Norm.Cyp19a1a.Eef1aG6pd, K.gonads.raw$Group, method = "bh")dunnNorm.Cyp19a1a.Eef1aG6pddunnNorm.Cyp19a1a.Eef1aG6pd = dunnNorm.Cyp19a1a.Eef1aG6pd$res#Letterslibrary("rcompanion")#Saves lettersletter_labels <- cldList(comparison = dunnNorm.Cyp19a1a.Eef1aG6pd$Comparison, p.value = dunnNorm.Cyp19a1a.Eef1aG6pd$P.adj, threshold  = 0.05)#Pulling out sex and group from the dataframe, pulling out the unique group/sex combosrosetta <- subset(K.gonads.raw,select=c("Group","Sex"))rosetta <- rosetta[with(rosetta,order(Group)),]rosetta <- unique(rosetta)#Ordering by group and binding to letter_labels dataframerosetta <- rosetta[with(rosetta,order(Group)),]rosetta <- cbind(letter_labels,rosetta)#Detaching the raw data so can attach the relative datadetach("K.gonads.raw")#Attaching relative data - for graphingK.gonads.rel <- read.csv("K.gonads.rel.csv", header=T)attach(K.gonads.rel)  #allows directly naming variablesnames(K.gonads.rel)  #gives an overview of variable names in the datasetK.gonads.rel$Group <- as.factor(K.gonads.rel$Group)library(ggplot2)#Creating an array so letter labels are 0.2 above the max value for each sexNorm.Cyp19a1a.Eef1aG6pd_ylabelpos <- NULLfor (i in rosetta$Group) {  subsetis <- (K.gonads.rel[which(K.gonads.rel$Group==i),])  Norm.Cyp19a1a.Eef1aG6pd_ylabelpos <- c(Norm.Cyp19a1a.Eef1aG6pd_ylabelpos,max(subsetis$Norm.Rel.Cyp19a1a.Eef1aG6pd))}Norm.Cyp19a1a.Eef1aG6pd_ylabelpos <- Norm.Cyp19a1a.Eef1aG6pd_ylabelpos+0.2#Graphmy_y_title <- expression(paste("Relative normalised ", italic(" cyp19a1a"), " expression"))ggplot(K.gonads.rel, aes(x = Sex, y = Norm.Rel.Cyp19a1a.Eef1aG6pd)) +  theme (panel.background = element_rect(fill = 'white', colour = 'white'))+  theme (axis.line.x = element_line(colour = 'black'))+  theme (axis.line.y = element_line(colour = 'black'))+  theme (axis.text.x = element_text(size=12, angle=45, hjust=1))+  theme (axis.text.y = element_text(size=12))+  theme (axis.title.x = element_text(size=12))+  theme (axis.title.y = element_text(size=12))+  geom_boxplot(outlier.size = NA)+  geom_jitter(width = 0.2, show.legend = FALSE, pch = 21, size = 2, fill = 'grey')+  scale_x_discrete(limits=c ("NBF", "ET", "TP", "IP"))+  scale_y_continuous(name=my_y_title)+  annotate("text", x = rosetta$Sex, y = Norm.Cyp19a1a.Eef1aG6pd_ylabelpos, label = rosetta$Letter)#If kruskall-wallis significant but dunns finds no pairwise significance change label = rosetta$Letter to label = "a"#saving graph with no y axis limitsmy_y_title <- expression(paste("Relative normalised ", italic("Cyp19a1a"), " expression"))#saving graph as pdf and with y axis limitsmy_y_title <- expression(paste("Relative normalised ", italic("cyp19a1a"), " expression"))pdf (file="relative.Norm.Cyp19a1a.Eef1aG6pd.pdf", width=8, height=4, par(lwd=0.01), paper="a4")ggplot(K.gonads.rel, aes(x = Sex, y = Norm.Rel.Cyp19a1a.Eef1aG6pd)) +  theme (panel.background = element_rect(fill = 'white', colour = 'white'))+  theme (axis.line.x = element_line(colour = 'black'))+  theme (axis.line.y = element_line(colour = 'black'))+  theme (axis.text.x = element_text(size=12, angle=45, hjust=1))+  theme (axis.text.y = element_text(size=12))+  theme (axis.title.x = element_text(size=12))+  theme (axis.title.y = element_text(size=12))+  geom_boxplot(outlier.size = NA)+  geom_jitter(width = 0.2, show.legend = FALSE, pch = 21, size = 2, fill = 'Gold2')+  scale_x_discrete(limits=c ("NBF", "ET", "TP", "IP"))+  scale_y_continuous(name=my_y_title, breaks = seq(0, 5, 1))+  coord_cartesian(ylim = c(0, 5))+  annotate("text", x = rosetta$Sex, y = Norm.Cyp19a1a.Eef1aG6pd_ylabelpos, label = rosetta$Letter)dev.off()#fill = 'Gold2' if overall significant, fill = 'grey' if not significant.#Example R code for statistical analysis and graphical illustration of NORMALISED qPCR data#load dataK.gonads.raw <- read.csv("K.gonads.raw.csv", header=T)attach(K.gonads.raw)  #allows directly naming variablesnames(K.gonads.raw)  #gives overview of variable names in datasetK.gonads.raw$Group <- as.factor(K.gonads.raw$Group)#Kruskal Wallis testkruskal.test(Cyp19a1a~Group, data = K.gonads.raw)#Dunn test with BH correctionlibrary("dunn.test")library("FSA")dunnCyp19a1a <- dunnTest(K.gonads.raw$Cyp19a1a, K.gonads.raw$Group, method = "bh")dunnCyp19a1adunnCyp19a1a = dunnCyp19a1a$res#Letterslibrary("rcompanion")#Saves lettersletter_labels <- cldList(comparison = dunnCyp19a1a$Comparison, p.value = dunnCyp19a1a$P.adj, threshold  = 0.05)#Pulling out sex and group from the dataframe, pulling out the unique group/sex combosrosetta <- subset(K.gonads.raw,select=c("Group","Sex"))rosetta <- rosetta[with(rosetta,order(Group)),]rosetta <- unique(rosetta)#Ordering by group and binding to the letter_labels dataframerosetta <- rosetta[with(rosetta,order(Group)),]rosetta <- cbind(letter_labels,rosetta)#Detaching the raw data so can attach the relative datadetach("K.gonads.raw")#Attach relative data - for graphingK.gonads.rel <- read.csv("K.gonads.rel.csv", header=T)attach(K.gonads.rel)  #allows directly naming variablesnames(K.gonads.rel)  #gives an overview of variable names in the datasetK.gonads.rel$Group <- as.factor(K.gonads.rel$Group)library(ggplot2)#Creating an array so lettler labels are 0.2 above the max value for each sexCyp19a1a_ylabelpos <- NULLfor (i in rosetta$Group) {    subsetis <- (K.gonads.rel[which(K.gonads.rel$Group==i),])    Cyp19a1a_ylabelpos <- c(Cyp19a1a_ylabelpos,max(subsetis$Rel.Cyp19a1a))}Cyp19a1a_ylabelpos <- Cyp19a1a_ylabelpos+0.2#Graphmy_y_title <- expression(paste("Relative ", italic("cyp19a1a"), " expression"))ggplot(K.gonads.rel, aes(x = Sex, y = Rel.Cyp19a1a)) +theme (panel.background = element_rect(fill = 'white', colour = 'white'))+theme (axis.line.x = element_line(colour = 'black'))+theme (axis.line.y = element_line(colour = 'black'))+theme (axis.text.x = element_text(size=16, angle=45, hjust=1))+theme (axis.text.y = element_text(size=14))+theme (axis.title.x = element_text(size=25))+theme (axis.title.y = element_text(size=16))+geom_boxplot(outlier.size = NA)+geom_jitter(width = 0.2, show.legend = FALSE, pch = 21, size = 2, fill = 'grey')+scale_x_discrete(limits=c ("NBF", "ET", "TP", "IP"))+scale_y_continuous(name=my_y_title)+annotate("text", x = rosetta$Sex, y = Cyp19a1a_ylabelpos, label = rosetta$Letter)# If kruskall-wallis significant but dunns finds no pairwise significance change label = rosetta$Letter to label = "a"#saving graph as pdf and with y axis limitsmy_y_title <- expression(paste("Relative ", italic("cyp19a1a"), " expression"))pdf (file="relative.Cyp19a1a.pdf", width=8, height=4, par(lwd=0.01), paper="a4")ggplot(K.gonads.rel, aes(x = Sex, y = Rel.Cyp19a1a)) +theme (panel.background = element_rect(fill = 'white', colour = 'white'))+theme (axis.line.x = element_line(colour = 'black'))+theme (axis.line.y = element_line(colour = 'black'))+theme (axis.text.x = element_text(size=16, angle=45, hjust=1))+theme (axis.text.y = element_text(size=14))+theme (axis.title.x = element_text(size=25))+theme (axis.title.y = element_text(size=14))+geom_boxplot(outlier.size = NA)+geom_jitter(width = 0.2, show.legend = FALSE, pch = 21, size = 2, fill = 'Gold2')+scale_x_discrete(limits=c ("NBF", "ET", "TP", "IP"))+scale_y_continuous(name=my_y_title, breaks = seq(0, 5, 1))+coord_cartesian(ylim = c(0, 5))+annotate("text", x = rosetta$Sex, y = Cyp19a1a_ylabelpos, label = rosetta$Letter)dev.off()#fill = 'Gold2' if overall significant, fill = 'grey' if not significant.#UN-NORMALISED#Example R code for statistical analysis and graphical illustration of NORMALISED qPCR data#load dataK.gonads.raw <- read.csv("K.gonads.raw.csv", header=T)attach(K.gonads.raw)  #allows directly naming variablesnames(K.gonads.raw)  #gives overview of variable names in datasetK.gonads.raw$Group <- as.factor(K.gonads.raw$Group)#Kruskal Wallis testkruskal.test(Cyp19a1a~Group, data = K.gonads.raw)#Dunn test with BH correctionlibrary("dunn.test")library("FSA")dunnCyp19a1a <- dunnTest(K.gonads.raw$Cyp19a1a, K.gonads.raw$Group, method = "bh")dunnCyp19a1adunnCyp19a1a = dunnCyp19a1a$res#Letterslibrary("rcompanion")#Saves lettersletter_labels <- cldList(comparison = dunnCyp19a1a$Comparison, p.value = dunnCyp19a1a$P.adj, threshold  = 0.05)#Pulling out sex and group from the dataframe, pulling out the unique group/sex combosrosetta <- subset(K.gonads.raw,select=c("Group","Sex"))rosetta <- rosetta[with(rosetta,order(Group)),]rosetta <- unique(rosetta)#Ordering by group and binding to the letter_labels dataframerosetta <- rosetta[with(rosetta,order(Group)),]rosetta <- cbind(letter_labels,rosetta)#Detaching the raw data so can attach the relative datadetach("K.gonads.raw")#Attach relative data - for graphingK.gonads.rel <- read.csv("K.gonads.rel.csv", header=T)attach(K.gonads.rel)  #allows directly naming variablesnames(K.gonads.rel)  #gives an overview of variable names in the datasetK.gonads.rel$Group <- as.factor(K.gonads.rel$Group)library(ggplot2)#Creating an array so lettler labels are 0.2 above the max value for each sexCyp19a1a_ylabelpos <- NULLfor (i in rosetta$Group) {  subsetis <- (K.gonads.rel[which(K.gonads.rel$Group==i),])  Cyp19a1a_ylabelpos <- c(Cyp19a1a_ylabelpos,max(subsetis$Rel.Cyp19a1a))}Cyp19a1a_ylabelpos <- Cyp19a1a_ylabelpos+0.2#Graphmy_y_title <- expression(paste("Relative ", italic("cyp19a1a"), " expression"))  ggplot(K.gonads.rel, aes(x = Sex, y = Rel.Cyp19a1a)) +  theme (panel.background = element_rect(fill = 'white', colour = 'white'))+  theme (axis.line.x = element_line(colour = 'black'))+  theme (axis.line.y = element_line(colour = 'black'))+  theme (axis.text.x = element_text(size=16, angle=45, hjust=1))+  theme (axis.text.y = element_text(size=14))+  theme (axis.title.x = element_text(size=25))+  theme (axis.title.y = element_text(size=16))+  geom_boxplot(outlier.size = NA)+  geom_jitter(width = 0.2, show.legend = FALSE, pch = 21, size = 2, fill = 'grey')+  scale_x_discrete(limits=c ("NBF", "ET", "TP", "IP"))+  scale_y_continuous(name=my_y_title)+  annotate("text", x = rosetta$Sex, y = Cyp19a1a_ylabelpos, label = rosetta$Letter)# If kruskall-wallis significant but dunns finds no pairwise significance change label = rosetta$Letter to label = "a"#saving graph as pdf and with y axis limitsmy_y_title <- expression(paste("Relative ", italic("cyp19a1a"), " expression"))pdf (file="relative.Cyp19a1a.pdf", width=8, height=4, par(lwd=0.01), paper="a4")ggplot(K.gonads.rel, aes(x = Sex, y = Rel.Cyp19a1a)) +  theme (panel.background = element_rect(fill = 'white', colour = 'white'))+  theme (axis.line.x = element_line(colour = 'black'))+  theme (axis.line.y = element_line(colour = 'black'))+  theme (axis.text.x = element_text(size=16, angle=45, hjust=1))+  theme (axis.text.y = element_text(size=14))+  theme (axis.title.x = element_text(size=25))+  theme (axis.title.y = element_text(size=14))+  geom_boxplot(outlier.size = NA)+  geom_jitter(width = 0.2, show.legend = FALSE, pch = 21, size = 2, fill = 'Gold2')+  scale_x_discrete(limits=c ("NBF", "ET", "TP", "IP"))+  scale_y_continuous(name=my_y_title, breaks = seq(0, 5, 1))+  coord_cartesian(ylim = c(0, 5))+  annotate("text", x = rosetta$Sex, y = Cyp19a1a_ylabelpos, label = rosetta$Letter)dev.off()#fill = 'Gold2' if overall significant, fill = 'grey' if not significant.
